# In Vitro Anti-Proliferative and Apoptotic Effects of Hydroxytyrosyl Oleate on SH-SY5Y Human Neuroblastoma Cells

**DOI:** 10.3390/ijms232012348

**Published:** 2022-10-15

**Authors:** Valentina Laghezza Masci, Roberta Bernini, Noemi Villanova, Mariangela Clemente, Vittoria Cicaloni, Laura Tinti, Laura Salvini, Anna Rita Taddei, Antonio Tiezzi, Elisa Ovidi

**Affiliations:** 1 Department for Innovation in Biological, Agro-Food and Forest Systems (DIBAF), University of Tuscia, Largo dell’Università, 01100 Viterbo, Italy; 2 Department of Agriculture and Forest Sciences (DAFNE), University of Tuscia, Via San Camillo de Lellis snc, 01100 Viterbo, Italy; 3 Toscana Life Science Foundation, Via Fiorentina 1, 53100 Siena, Italy; 4 High Equipment Centre, Tuscia University, Largo dell’Università snc, 01100 Viterbo, Italy

**Keywords:** plant-derived compounds, hydroxytyrosol, hydroxytyrosol oleate, SH-SY5Y human neuroblastoma cells, cytotoxicity, anti-proliferative activity, apoptosis induction activity

## Abstract

The antitumor activity of polyphenols derived from extra virgin olive oil and, in particular the biological activity of HTyr, has been studied extensively. However, the use of HTyr as a therapeutic agent for clinical applications is limited by its low bioavailability and rapid excretion in humans. To overcome these limitations, several synthetic strategies have been optimized to prepare lipophenols and new compounds derived from HTyr to increase lipophilicity and bioavailability. One very promising ester is hydroxytyrosyl oleate (HTyr-OL) because the chemical structure of HTyr, which is responsible for several biological activities, is linked to the monounsaturated chain of oleic acid (OA), giving the compound high lipophilicity and thus bioavailability in the cellular environment. In this study, the in vitro cytotoxic, anti-proliferative, and apoptotic induction activities of HTyr-OL were evaluated against SH-SY5Y human neuroblastoma cells, and the effects were compared with those of HTyr and OA. The results showed that the biological activity of HTyr was maintained in HTyr-OL treatments at lower dosages. In addition, the shotgun proteomic approach was used to study HTyr-OL-treated and untreated neuroblastoma cells, revealing that the antioxidant, anti-proliferative and anti-inflammatory activities of HTyr-OL were observed in the unique proteins of the two groups of samples.

## 1. Introduction

The biological activities of natural substances have been exploited since ancient times and have been used to treat various human diseases [1]. The relevant anti-tumor properties of some plant molecules and semi-synthetic or synthetic analogues are well studied and are used against several types of cancer today [2,3,4].

**Figure 1:**
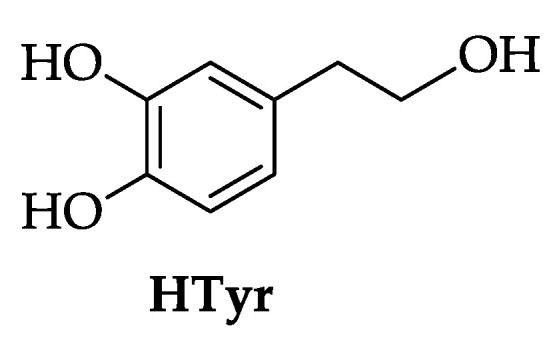
Chemical structure of hydroxytyrosol (HTyr).

Hydroxytyrosol (3,4-dihydroxyphenethyl alcohol, HTyr, Figure 1) is a low-molecular-weight and amphiphilic phenol found in the fruit of the olive tree (*Olea europaea* L.), a plant widely cultivated in Spain, Italy, Greece, and Turkey either for the consumption of table olives or to produce extra-virgin olive oil (EVOO).

EVOO represents a key component of the Mediterranean diet, a dietary regime which recommends the daily intake of vegetables, fruits, grains, legumes, and nuts for a healthy lifestyle and to prevent the onset of cardiovascular diseases, cancer, type 2 diabetes, neurodegenerative disorders, and aging [5,6]. These foods are rich in active ingredients, including phenolic compounds, which are responsible for their beneficial effects on human health [7,8].

During olive ripening and EVOO production, the content of HTyr in fruit increases due to the hydrolytic activity of an endogenous β-glucosidase on oleuropein, a secoiridoid derivative, giving rise to HTyr, elenolic acid, and glucose [9].

Both oleuropein and HTyr can be recovered from olive oil by-products by innovative and eco-friendly technologies, according to the modern “circular economy strategy”, in which waste from one production process represents the raw materials for a new process [10]. Oleuropein can be extracted from olive leaves, a solid waste derived mainly from the pruning of olive trees, while HTyr can be recovered from liquid (olive oil wastewaters) and solid (pomace) wastes produced during EVOO processing [11,12].

In vitro experiments have evidenced that HTyr is not genotoxic and mutagenic at concentrations corresponding to the daily consumption of EVOO [13]. In vivo studies have shown that HTyr does not induce effects of toxicological significance even at high doses with a proposed NOAL (No Observed Adverse Effect Level) of 500 mg/kg/d [14].

HTyr displays a variety of biological activities [15], including antioxidant [16], anti-inflammatory [17], anticancer [18,19,20], anti-diabetic [21], cardioprotective [22], and neuroprotective [23,24] properties. Recent studies have evidenced that HTyr plays a relevant role in the prevention of neurodegenerative diseases [25], in the neurogenesis of adult and aged mice and in increasing the survival of new neurons [26].

For all its described properties, HTyr has been granted the Generally Recognized as Safe (GRAS) designation by the United States Food and Drug Administration (FDA), and is a precious ingredient for nutraceutical, cosmeceutical, and food formulations [13].

For its content in the EVOO, the European Food Safety Authority (EFSA) has approved a health claim which states that “olive oil polyphenols contribute to the protection of blood lipids from oxidative stress”. However, this effect is observed only with a daily intake of 20 g of an olive oil “containing at least 5 mg of hydroxytyrosol and its derivatives (e.g., oleuropein complex and tyrosol)” [27].

Despite both its excellent safety profile and strong biological activities, the use of HTyr as a therapeutic agent for clinical applications is limited by its low bioavailability and fast excretion in humans [28].

To overcome these limitations, a variety of synthetic strategies have been optimized to prepare lipophenols as HTyr esters [29,30,31,32], HTyr ethers [33,34,35], and new HTyr-derived compounds [36,37,38].

Among them, HTyr alkyl esters are the most studied compounds. Structurally, they are characterized by the catecholic moiety, as well as an alkyl chain linked by an ester bond on the alcoholic functionality. The chain length allows for modulation of the lipophilicity, a property that modifies the bioavailability of a molecule in the cellular environment and its solubility in non-aqueous media as oils and emulsions [32,39].

**Figure 2:**
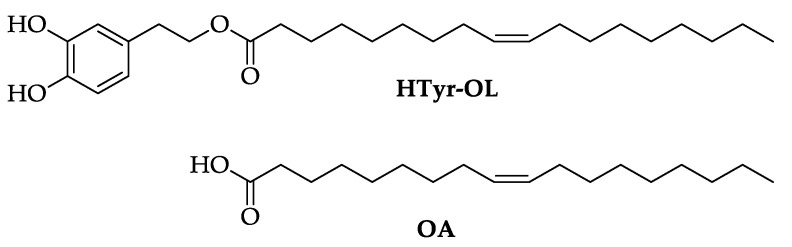
Chemical structure of hydroxytyrosyl oleate (HTyr-OL) and oleic acid (OA).

A very promising ester is hydroxytyrosyl oleate (HTyr-OL), a compound characterized by a high lipophilicity due to the presence of the C18 monounsaturated chain of oleic acid (OA, Figure 2). Recently, HTyr-OL has been identified in EVOO and olive oil by-products, while it has been found to be absent in olives, suggesting that it is obtained during oil production [40].

In recent years, HTyr-OL has aroused interest; however, only a few studies have been published to date. HTyr-OL has shown an anti-proliferative effect on the human colon cancer cell line HCT8-β8, engineered to overexpress oestrogen receptor β (ERβ) [41]; exhibited anti-inflammatory activity by reducing nitric oxide production by lipopolysaccharide-stimulated RAW264.7 macrophages [40]; antioxidant activity in human keratinocytes [42]; anti-diabetic properties by inducing insulin secretion in pancreatic cell line 832/13 [43].

To the best of our knowledge, no studies have yet been conducted on the effects of HTyr-OL on the SH-SY5Y human neuroblastoma cell line. Neuroblastoma is a pediatric cancer arising from neural progenitor cells with a very high biological and clinical heterogeneity [44].

Due to the lack of literature, the aim of this investigation was to evaluate the in vitro cytotoxicity, anti-proliferative, and apoptotic induction activities of HTyr-OL against SH-SY5Y human neuroblastoma cells, and to then compare these effects to those of HTyr and OA separately. The cell viability assays were followed by Western blot, flow cytometry, electron microscopy and proteomic techniques. The proteomic approach was applied downstream to analyse the cellular pathways involved in the response to HTyr-OL treatment.

## 2. Results

### 2.1. Synthesis of HTyr and HTyr-OL

**Scheme 1:**
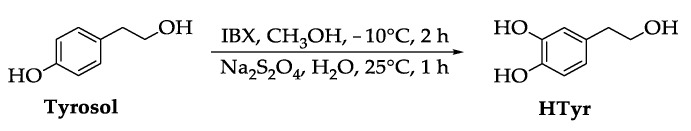
Synthesis of HTyr.

HTyr was synthetized according to a procedure already reported in the literature [45], with some modifications. Briefly, tyrosol (4-hydroxyphenethyl alcohol) was oxidized with 2-iodoxybenzoic acid [1-hydroxy-1-oxo-1H-1λ5- benz[d][1,2]iodoxol-3-one, IBX] [46] in methanol and then treated with sodium dithionite (Na_2_S_2_O_4_) in water. After chromatographic purification, HTyr was isolated in 40% yield (Scheme 1).

**Scheme 2:**
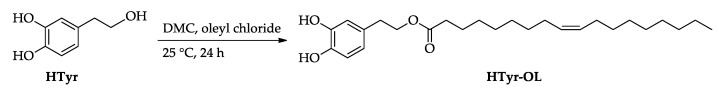
Synthesis of HTyr-OL.

HTyr-OL was obtained by esterification of HTyr with oleyl chloride in dimethyl carbonate (DMC), as depicted in Scheme 2. HTyr-OL was isolated in a 60% yield [32].

### 2.2. Cytotoxic Activity

**Figure 3:**
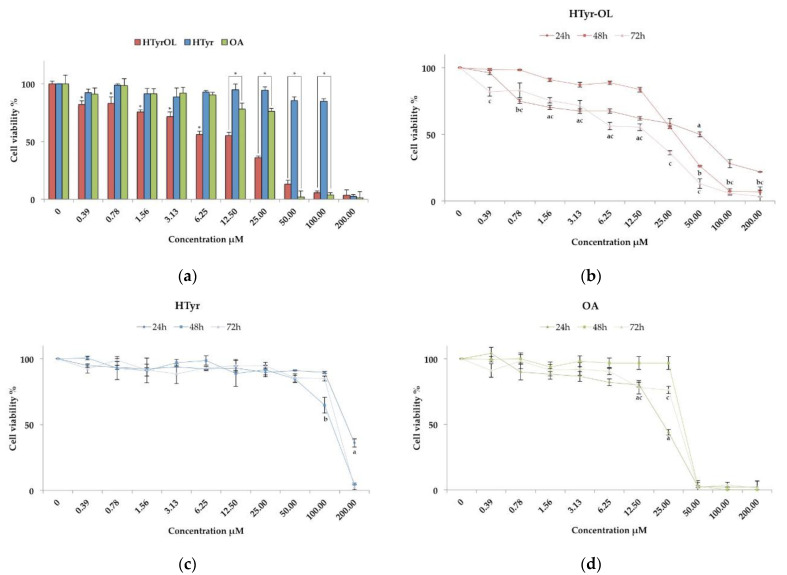
(**a**) Graph bar of cytotoxic activity. The cell viability percentages of SH-SY5Y are plotted against treatment concentrations; (**b**) Line graphs of the anti-proliferative effects of HTyr-OL, (**c**) HTyr, and (**d**) OA on SH-SY5Y neuroblastoma cells at 24, 48, and 72 h. Results are expressed as mean of cell viability against concentration (µM). The values are expressed as % ± SD. *, *p*-value < 0.05 significant differences compared with ctrl group; a, *p*-value < 0.05 significant differences compared with 48 and 72 h; b, *p*-value < 0.05 significant differences compared with 24 and 72 h; c, *p*-value < 0.05 significant differences compared with 24 and 48 h.

The cytotoxic effect of HTyr-OL, HTyr and OA on SH-SY5Y cells was evaluated using the MTT assay, and cell viability was measured after 72 h of treatment. The results showed that HTyr-OL had a stronger dose-dependent effect on neuroblastoma cells than HTyr and OA (Figure 3a).

After 72 h of exposure to the treatments, by comparing the half maximal effective concentration (EC_50_) values obtained for SH-SY5Y cells, the activity of HTyr-OL was found to be stronger than that of HTyr. The EC_50_ value of HTyr, 114.02 ± 1.69 µM, was about four times higher than that of HTyr-OL, 26.35 ± 3.75 µM. The OA-treated cells responded with an EC_50_ value of 35.93 ± 3.57 µM.

To assess whether the observed cytotoxic activity may be selective on tumor cells, normal human cell lines (MCF10A) were treated as the SH-SY5Y cells; EC_50_ values above 200 µM were obtained for all three compounds (Table 1), thus confirming a more selective affection of HTyr-OL to the neuroblastoma cell model.

### 2.3. Anti-Proliferative Effect

The anti-proliferative effects of HTyr-OL, HTyr, and OA were studied by measuring the mitochondrial dehydrogenase activity as an indicator of the cell viability (MTT assay) of SH-SY5Y cells after 24, 48, and 72 h of treatment exposure. HTyr-OL demonstrated dose- and time-dependent effects on neuroblastoma cells (Figure 3b), while HTyr and OA showed dose-dependent and non-time-dependent effects (Figure 3c,d). This evidence was also provided by the EC_50_ values obtained.

As depicted in Table 2, the EC_50_ values of HTyr-OL decreased by about half as the treatment time increased from 24 to 48 h, ranging from 48.97 ± 9.04 µM to 27.76 ± 2.31 µM. The growth inhibition obtained at 48 h was maintained at 72 h, whereas the EC_50_ values showed no significant change.

### 2.4. Apoptosis Detection by Flow Cytometry

**Figure 4:**
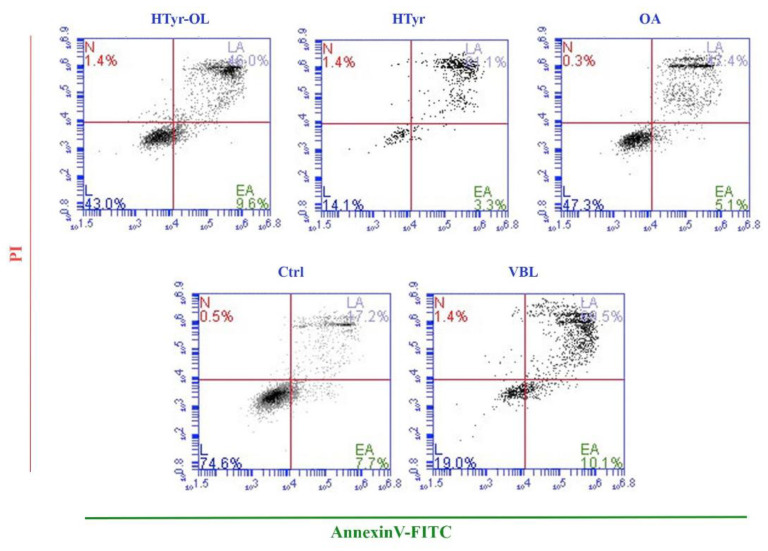
Apoptosis detection by flow cytometry. Flow cytometric analysis of the HTyr-OL, HTyr and OA treatments on SH-SY5Y cells for apoptosis determination using AnnexinV-FITC/PI staining. L: live cells; EA: early apoptotic cells; LA: late apoptotic cells; N: necrotic cells.

The cytotoxic and anti-proliferative effects observed following treatments with HTyr-OL, HTyr, and OA were further investigated to determine whether cell death was related to an apoptotic process. Neuroblastoma cells were treated for 24 h with EC_50_ doses of the tested compounds and processed for flow cytometry, with the presence of Annexin V-FITC and Propidium Iodide (PI) being analyzed (Figure 4).

The cells treated with HTyr-OL and OA showed similar apoptotic rate percentages, 52.36% ± 4.31% and 49.91% ± 6.94%, respectively, corresponding to the sum of the percentage of Annexin V-positive/PI-negative stained (early apoptosis) and Annexin V-positive/PI-positive (late apoptosis) stained cells. The highest apoptotic rate was found for the HTyr-treated cells (79.64% ± 8.83%), with a value close to that of vinblastine treated (VBL treated) cells (74.24% ± 6.29%) obtained (Table 3).

### 2.5. Scanning Electron Microscopy Investigation

**Figure 5:**
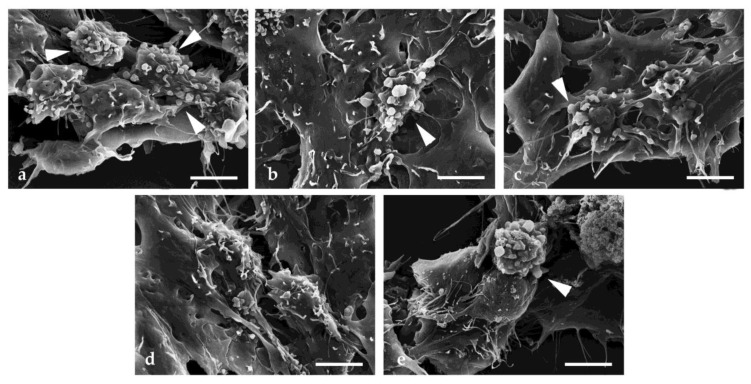
SEM micrographs of SH-SY5Y cells at 24 h of EC_50_ treatments, with: (**a**) HTyr-OL; (**b**) HTyr; (**c**) OA; (**d**) Ctrl or untreated cells; (**e**) VBL. Arrowheads for membrane blebbing of apoptotic cells. Scale bars 5 µm.

To define the morphological changes following the treatments with HTyr-OL, HTyr, and OA in SH-SY5Y cells, scanning electron microscopy was performed. Treatment with HTyr-OL resulted in a change in cell morphology, with the appearance of spherical-shaped cells and subsequent loss of cell adhesion observed (Figure 5a). The morphologically altered cells were characterized by surface changes, with the presence of protrusions and blebs observed. The same conditions at the plasma membrane level were found in HTyr and OA treated cells (Figure 5b,c), in which no rounded cells with a loss of adhesion were detected. In Figure 5d, Ctrl cells are shown that were characterized by a normal morphology, and a regular surface with thin and properly distributed cell protrusions, typical of an adherent cell. In contrast, SH-SY5Y treated with VBL showed typically apoptotic features, with the presence of roundish cells, morphological alterations, and blebs on the surface observed (Figure 5e).

### 2.6. Detection of Caspase-3 Activated by Western Blot

To validate the results obtained by flow cytometry, the presence of activated caspase-3 was detected by immunoblotting techniques. The monoclonal antibody against activated caspase-3 recognized two bands (19 kDa and 17 kDa), corresponding to the major and minor fragments, resulting in the cutting of the amino acid Asp175 in caspase-3.

**Figure 6:**
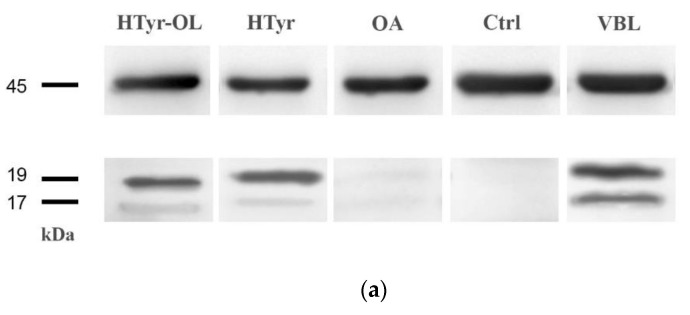

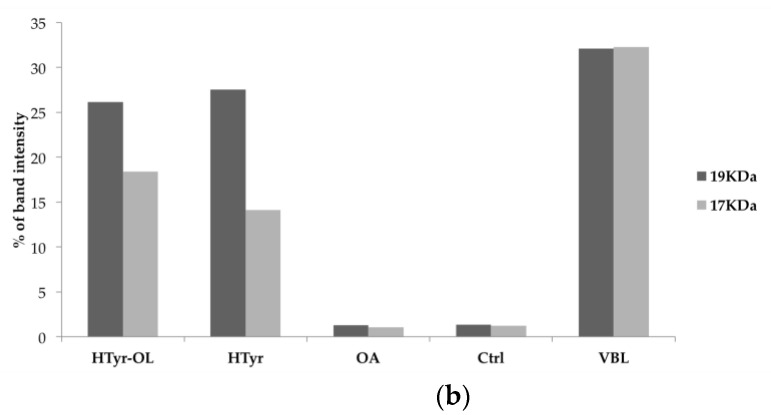
Western blot analysis for expression of cleaved caspase-3, studied using a cleaved caspase-3 monoclonal antibody. (**a**) In the lower panel, the representative image of detection of cleaved caspase-3 in SH-SY5Y cells treated with HTyr-OL, HTyr, OA; Ctrl and VBL cells were negative and positive controls, respectively (one of the three experiments). In the upper panel, detection of β-actin as the internal control; (**b**) Graph bar showing differences in intensity of caspase-3 obtained by pictures in (**a**), quantitatively evaluated using the ImageJ program.

Figure 6a shows that in HTyr-OL and HTyr-treated samples, both bands were detected, with intensities obtained that were comparable to those of samples treated with apoptosis inducer (VBL). In the lysates obtained in OA-treated cells and untreated cells (Ctrl), very weak bands were detected, the intensity of which was determined by image processing and subsequent quantification (Figure 6b).

### 2.7. Shotgun Proteomic Approach and Gene Ontology Functional Annotation

**Figure 7:**
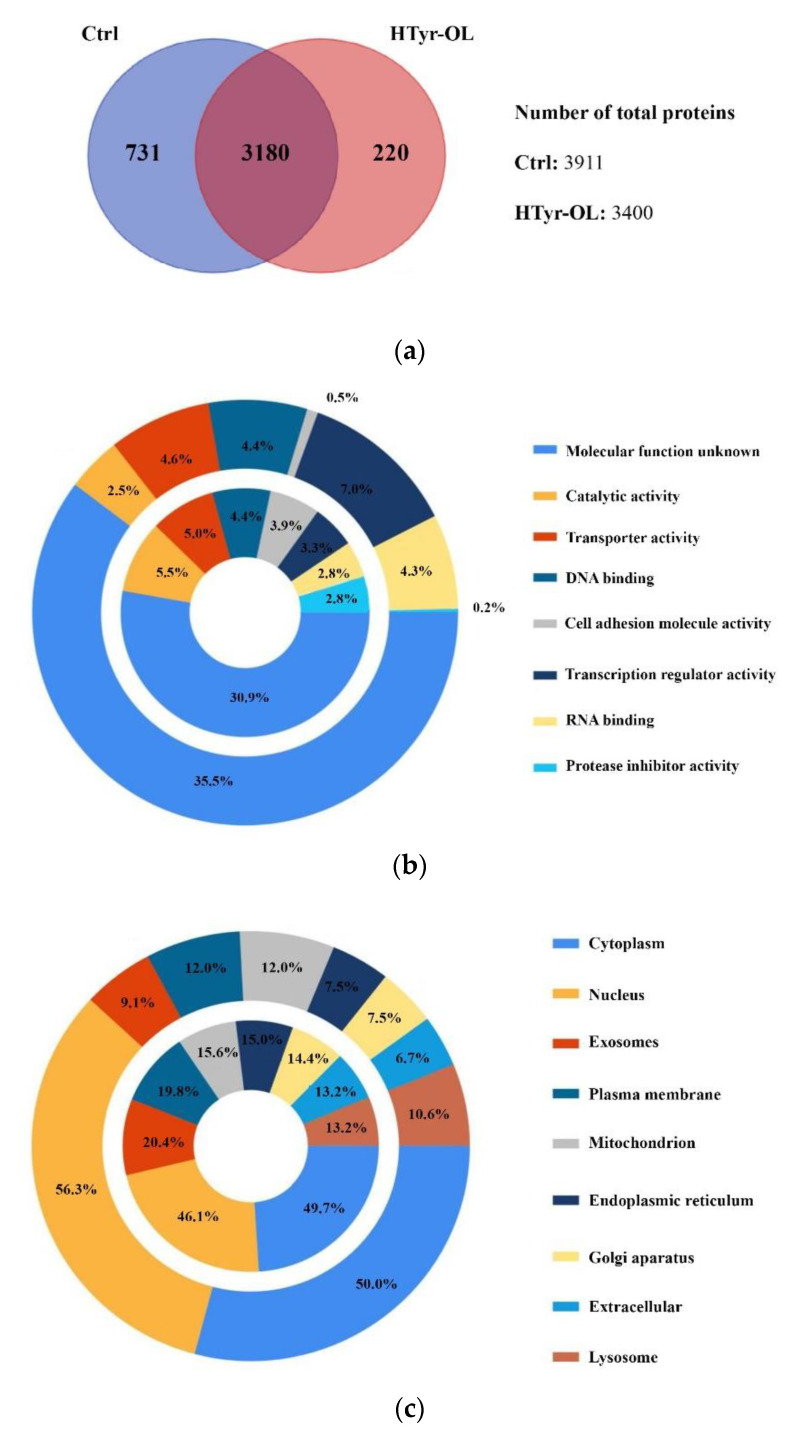
Identified proteins and Gene Ontology analysis. (**a**) Venn diagram representing the number of reproducibly quantified proteins for Ctrl (3911 total proteins) and HTyr-OL (3400 total proteins) samples. (**b**) Molecular functions comparison between Ctrl and HTyr-OL groups. (**c**) Cellular components comparison between Ctrl and HTyr-OL groups. In (**b**,**c**), Ctrl is on the outer chart and HTyr-OL is on the inner chart.

A shotgun proteomic approach was performed to investigate the differential protein expression profile in Ctrl and HTyr-OL samples. A total of 3911 and 3400 proteins were identified in the treatment group (HTyr-OL) and Ctrl group, respectively, deriving from the triplicates of each condition. A total of 3180 unique proteins associated with a known *Homo sapiens* protein were found to be shared by the two sample groups, 220 were unique in HTyr-OL and 731 were unique in the Ctrl group. A numerical comparison was carried out, and this is presented in a Venn diagram (Figure 7a). The “overlap index” based on the Jaccard similarity coefficient (which measures similarity between finite sample sets and is defined as the size of the intersection divided by the size of the union of the sample sets) was 0.77. For the Ctrl group, 18.69% of the total proteome was unique, while 81.31% was shared with HTyr-OL. Similarly, for the HTyr-OL sample, 6.47% of the whole protein dataset was unique, while 93.53% was shared with the Ctrl.

**Figure 8:**
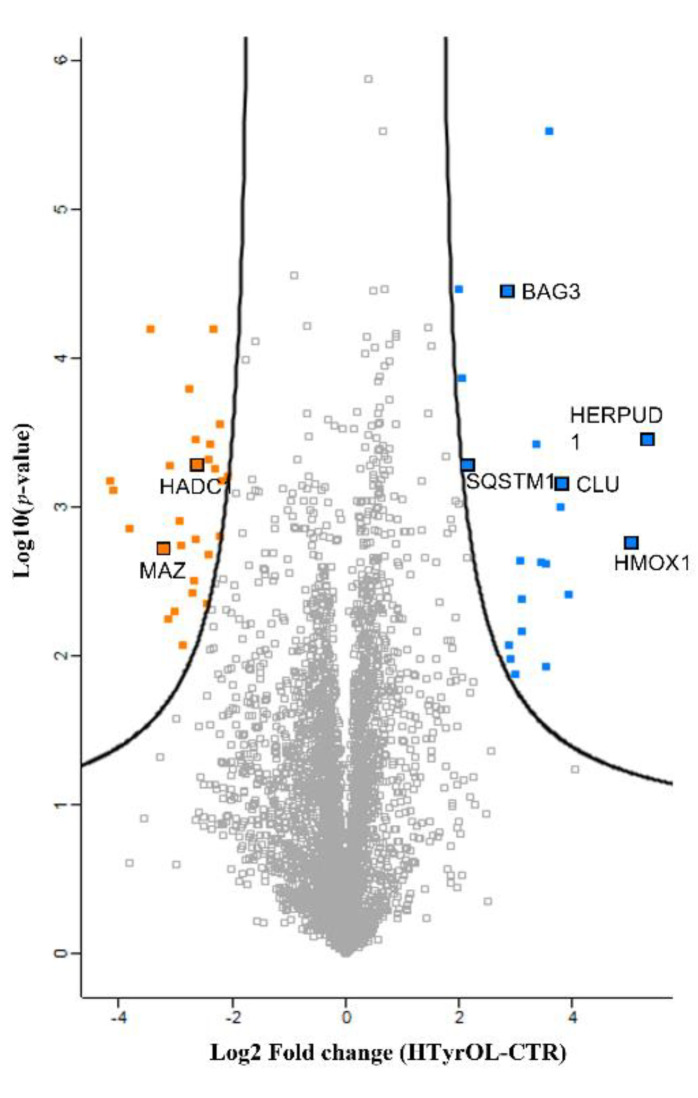
Volcano plot showing proteins differentially expressed in HTyr-OL and Ctrl datasets. Proteins with statistically significant differential expression (S0 = 2, *p*-value > 0.05) are in the top, right and left, quadrants. Upregulated proteins in HTyr-OL are represented in blue; downregulated proteins in HTyr-OL are in orange.

The unique protein lists identified in the Ctrl and HTyr-OL treated groups were subjected to functional annotation analysis. The enriched Gene Ontology (GO) terms in the molecular function (Figure 7b) category showed a significant difference (*p*-value < 0.001) in favour of Ctrl in GO terms “Transcription regulator activity” (7.0% in Ctrl; 3.3% in HTyr-OL) and “RNA binding” (4.3% in Ctrl; 2.8% in HTyr-OL), whereas the terms “Cell adhesion molecule activity” (0.5% in Ctrl; 3.9% in HTyr-OL) and “Protease inhibitor activity” (0.2% in Ctrl; 2.8% in HTyr-OL) were found to be enriched in HTyr-OL rather than in Ctrl. As far as the cellular component was concerned (Figure 8c), the GO terms “Exosomes” (9.1% in Ctrl; 20.4% in HTyr-OL), “Plasma membrane” (12.0% in Ctrl; 19.8% in HTyr-OL), “Endoplasmic Reticulum” (7.5% in Ctrl; 15.0% in HTyr-OL), “Golgi apparatus” (7.5% in Ctrl; 14.4% in HTyr-OL) and “Extracellular” (6.7% in Ctrl; 13.2% in HTyr-OL) appeared to be significantly enriched GO terms in the HTyr-OL group rather than in Ctrl. In particular, there was a high percentage of unique Ctrl proteins involved in cellular proliferation pathways, such as “Neuroblast proliferation” (67.55% in Ctrl; 32.45% in HTyr-OL), in the inflammation process, such as “Cytokine production involved in inflammatory response” (73.51% in Ctrl; 26.49% in HTyr-OL), “Stress-activated MAPK cascade” (89.28% in Ctrl; 10.72% in HTyr-OL) and “Stress-activated protein kinase signalling cascade” (90.02% in Ctrl; 9.98% in HTyr-OL) and in antioxidant activity, such as “Cellular response to oxidative stress” (60.05% in Ctrl; 39.95% in HTyr-OL).

Based on these findings, we investigated also the up/downregulated proteins and specifically selected those involved in apoptosis and proliferation processes. In Figure 8, a Volcano plot comparing the HTyr-OL dataset to the Ctrl dataset is presented; we found a total of 38 statistically significant proteins with up/downregulation. The DEPs identified had ratios higher than 2 and lower than −2, and they were grouped as upregulated and downregulated proteins, respectively. Among these proteins, 17 were found to be upregulated in HTyr-OL and 21 were found to be downregulated (for a complete list, see Table S1 in Supplementary Materials). These DEPs were then classified into their respective protein classes by mapping them into the Uniprot classification GO system [47] linked with apoptosis and cellular proliferation (five upregulated and two downregulated proteins).

#### Protein–Protein Interaction Network

**Figure 9:**
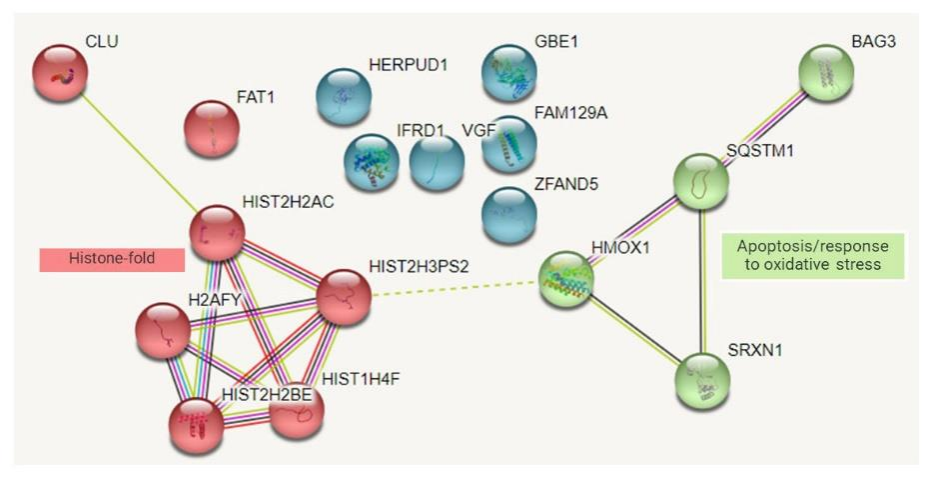
Protein–protein interaction (PPI) network of DEPs identified in HTyr-OL that describes the node proteins, wherein nodes in the same cluster are shown in the same color. The lines connecting the nodes indicate the associations between the proteins. The first cluster “Histone fold” is in red, and the second cluster “Apoptosis and response to oxidative stress” is in green. Proteins in blue are not included in a cluster.

The up- and downregulated protein DEPs were then classified and clustered into their respective protein classes by mapping them into the STRING classification system. The PPI network of all upregulated proteins in the HTyr-OL treated cells is presented in Figure 9. A total of 17 nodes and 13 edges were identified in the PPI network, and two significant interactions between DEPs were generated from k-means clustering analysis. Both networks also revealed that the DEPs with similar functional roles were grouped into a cluster, and inter-cluster interaction was displayed. The highly connected first cluster (in red in Figure 9) was made up of “Histone Fold” proteins (e.g., Core histone macro-H2A.1, H2AFY; Histone cluster 1 H4 family member f, HIST1H4F; Histone cluster 2 h2a family member c, HIST2H2AC; Histone cluster 2 h2b family member e, HIST2H2BE; Histone cluster 2 H3 pseudogene 2, HIST2H3PS2). The second cluster consisted of proteins involved in apoptosis and the response to oxidative stress, such as BAG3, SQSTM1, HMOX1, and Sulfiredoxin-1 (SRXN1). The remaining un-clustered proteins were 1,4-Alpha-glucan-branching enzyme (GBE1), HERPUD1, Interferon-related developmental regulator 1 (IFRD1), Neuro secretory protein VGF (VGF), AN1-type zinc finger protein 5 (ZFAND5), and Protein Niban 1 (FAM129A).

## 3. Discussion

HTyr-OL is a very interesting compound, having in its chemical structure the catechol moiety of HTyr, which is responsible for a variety of biological activities, linked to the monounsaturated chain of OA, which confers high lipophilicity and bioavailability in the cellular environment.

To investigate the potentiality of HTyr-OL to act on tumoral cell lines, this research was focused on the evaluation of HTyr-OL’s in vitro cytotoxicity, anti-proliferative, and apoptotic induction activities against SH-SY5Y human neuroblastoma cells. Its effects were compared to those of HTyr and OA.

For this purpose, HTyr and HTyr-OL were synthesized in our laboratory upon use to obtain samples of high purity, while OA was a commercially available product. HTyr-OL was prepared in a good yield (60%) by treating HTyr with oleyl chloride in DMC. Fresh HTyr was obtained in a moderate yield (40%) by the direct oxidation of tyrosol with IBX in methanol, and the following reductive step was carried out with Na_2_S_2_O_4_ in water [45,46]. Both products were purified by flash chromatography.

In our study, the cytotoxic and anti-proliferative assays demonstrated that the activity of HTyr against SH-SY5Y cells was enhanced in its lipophilic derivate HTyr-OL, which showed lower EC_50_ values over the three incubation times considered, as shown in Table 2. SH-SY5Y cells were also investigated by the flow cytometry technique and by Western blot analysis to search for the presence of Annexin V-FITC and Propidium Iodide (PI) staining and caspase-3, respectively, to determine whether the cell death was related to an apoptotic process. Apoptosis is a fundamental mechanism that enables the maintenance of the normal balance between cell death and survival to prevent cancer and other associated diseases [48].

The cytotoxicity induced by HTyr-OL on SH-SY5Y cells was clearly demonstrated to be related to an apoptotic process, with evidence provided by the apoptotic rate of 52.36 ± 4.31%, the presence of active caspase 3, and the clear morphological and ultrastructural changes observed, namely cell shrinking, the presence of rounded cells whose surfaces was characterized by apoptotic blebs [49].

The obtained results indicated that the anti-proliferative effects induced by HTyr-OL were increased compared with the HTyr and OA-treated cells. Cell growth inhibition by HTyr was demonstrated in several cancer cell lines, such as three colon carcinoma cell lines (HT-29, HCT-116, CT-26) [50]. In mammary adenocarcinoma cells (MCF-7) and in human leukaemia cells (HL-60), HTyr was found to have dose- and time-dependent anti-proliferative activity above 200 μM after 12 h of exposure for MCF-7 and at 100 μM after 24 h of exposure for HL-60; in both cases, the inhibition of cell growth was observed to be related to the induction of an apoptotic process [51,52].

The induction of apoptosis by HTyr was studied in pancreatic carcinoma cells (MIA PaCa-2) in which an apoptotic rate of 47.17% was reported, correlating with the detection of caspases 3 and 7 [53]. Furthermore, chemopreventive effects of HTyr due to the induction of anti-proliferative and apoptotic activity were observed in human promyelocytic cells (HL60) and in colon adenocarcinoma cells (HT-29 and DLD1) [54,55,56,57].

The results of the present study regarding the OA treatments revealed the positivity of Annexin V/IP by flow cytometry, coupled with the absence of cleaved caspase-3 detection by Western blot, confirming the data reported by Zhu and colleagues (2005), who observed an apoptotic process on neuronal cells through an OA-induced caspase-3-independent mechanism [58]. Leist and Jaattela in 2001 proposed the classification of different types of programmed cell deaths including an apoptosis-like process characterized by less compact chromatin condensation, phosphatidylserine exposure and absence of executioner caspase activation [59]. However, the mechanisms of OA-induced cell death are still unclear and should be further investigated, as Carrillo and colleagues (2012) concluded in a review on the antitumor effects of OA [60].

In the present study, the results showed that the anti-proliferative activity of HTyr-OL is due to a mechanism of cell death by apoptosis, an effect that was revealed at doses even four times lower than the doses of HTyr.

Lee Yee Qian et al. (2021) evaluated the activities of a curcumin derivative, demonstrating its potent dose dependent cytotoxicity, and time- and dose-dependent anti-proliferative activity in both U-87 MG and SH-SY5Y cells, performing better than its parent compound, curcumin. The authors, by using a proteomic analysis of these cells following treatment with MS13, found that several common Differential Expressed Proteins (DEPs) are involved in the regulation of specific pathways [61].

In the present study, the DEPs profiles, investigated in Ctrl and HTyr-OL samples by the shotgun proteomic approach, revealed that in the biological process category, the most significantly enriched GO terms annotated by the unique proteins in the two sample groups confirm the antioxidant, antiproliferative, and anti-inflammatory activities of HTyr-OL. Namely, a high percentage of unique Ctrl proteins involved in cellular proliferation pathways, such as “Neuroblast proliferation” (67.55% in Ctrl, 32.45% in HTyr-OL), in the inflammation process such as “Cytokine production involved in inflammatory response” (73.51% in Ctrl; 26.49% in HTyrOL), “Stress-activated MAPK cascade” (89.28% in Ctrl; 10.72% in HTyr-OL), “Stress-activated protein kinase signalling cascade” (90.02% in Ctrl; 9.98% in HTyr-OL) and in antioxidant activity, such as “Cellular response to oxidative stress” (60.05% in Ctrl; 39.95% in HTyr-OL), was found. Moreover, it should be noted that a high percentage of the Ctrl proteins were found to be involved in “Mitotic DNA damage checkpoint signalling” (61.82% in Ctrl; 38.18% in HTyr-OL) and “DNA damage checkpoint signalling” (69.81% in Ctrl; 30.19% in HTyr-OL). This aspect confirmed that DNA damage was more represented in the Ctrl group rather than in the HTyr-OL treated samples, which showed dose- and time-dependent antiproliferative and apoptotic effects.

Among the 220 HTyr-OL proteins, the “induced myeloid leukaemia cell differentiation protein Mcl-1” (MCL1, or its alternative name Bcl-2-like protein 3 (Bcl2-L-3) is involved in the regulation of apoptosis versus cell survival, and in the maintenance of viability but not proliferation. It mediates its effects by interactions with a number of other regulators of apoptosis, and it is implicated in the extrinsic apoptotic signalling pathway in the absence of ligands, and the intrinsic apoptotic signalling pathway in response to DNA damage [62]. Additionally, another protein, Presenilin 1 (PSEN1), is involved in cell proliferation inhibition and apoptosis induction by suppressing phosphatidylinositol 3-kinase/protein kinase B (PI3K/AKT) signalling [63,64,65].

Specifically, among the upregulated proteins, BAG family molecular chaperone regulator 3 (BAG3) is included. This is involved in the extrinsic apoptotic signaling pathway via death domain receptors [GO:0008625], which gather molecular signals conveyed from the cell surface to trigger the apoptotic death of a cell. The pathway starts with a ligand binding to a death domain receptor on the cell surface, and ends when the execution phase of apoptosis is triggered. Similarly, Heme oxygenase 1 (HMOX1), Clusterin (CLU), and Homocysteine-responsive endoplasmic reticulum-resident ubiquitin-like domain member 1 protein (HERPUD1) are connected to the intrinsic apoptotic signaling pathway in response to DNA damage [GO:0008630]. Such a pathway is induced by the detection of DNA damage and ends when the execution phase of apoptosis is triggered. In the positive regulation of the apoptotic process, the Sequestosome-1 (SQSTM1) protein is also implicated.

Conversely, among downregulated proteins, Myc-associated zinc finger protein (MAZ) is linked with the negative regulation of the apoptotic signaling pathway [GO:2001234], including any process that stops, prevents, or reduces the frequency, rate, or extent of the apoptotic signaling pathway. Another downregulated HTyr-OL protein is Histone deacetylase 1 (HDAC1), which is associated with the positive regulation of cell population proliferation [GO:0008284].

A histone fold is a structurally conserved motif found near the C-terminus in every core histone sequence in a histone octamer, and is responsible for the binding of histones into heterodimers. Histone-fold proteins are important in DNA damage repair [66].

## 4. Materials and Methods

### 4.1. Chemical and Reagents

Reagents, chemicals (including OA), solvents, silica gel 60 F254 plates, and silica gel 60 were furnished by Merck (Milan, Italy). HTyr and HTyr-OL were synthesized in laboratory as reported in Section 4.3 [45]. IBX was prepared upon use according to the procedure described in the literature [67].

### 4.2. Instrumentation

A 400 MHz Nuclear Magnetic Resonance Spectrometer Advance-III Bruker (Munich, Germany) was used to record the spectra (^1^H NMR, ^13^C NMR) of all compounds. Chemical shifts were expressed in parts per million (δ scale). Each sample (25–30 mg) was solubilized in chloroform-d3 or methanol-d4 (0.5 mL).

The absorbance of solubilized, purple-colored formazan product by MTT assay was read by a Tecan Sunrise™ UV-vis spectrophotometer at 595 nm. The emitted fluorescence of cells was stained with an Annexin V-FITC conjugate and PI, and was analysed using a flow cytometer (BD Accuri C6 Plus, BD Italy S.p.A, Milan, Italy). The samples for scanning electron microscopy analysis were dried with a critical point dryer using CO2 in a Balzers Union CPD 020, coated with gold in a Balzers MED 010 unit, and observed under a JEOL JSM 5200 electron microscope (Jeol Ltd., Tokyo, Japan).

LC-MS/MS analyses were performed using Q-Exactive HF-X UHPLC Nano mass spectrometer (Thermo Scientific, Waltham, MA, USA). The peptide separation was carried out at 37 °C using a peptide PepMap RSLC C18 column, 75 μm × 15 mm, 2 μm, 100 Å (Thermo Scientific, Waltham, MA, USA) at a flow rate of 0.300 μL/min.

### 4.3. Synthesis of HTyr and HTyr-OL

Tyrosol (138 mg, 1.0 mmol) was solubilized in methanol (10 mL) and kept at -10 °C. Then, IBX (403 mg, 1.44 mmol) was added under stirring. After 2 h, the mixture was placed at room temperature; then, water (3.5 mL) and Na_2_S_2_O_4_ (348 mg, 2.0 mmol) were added. After 1 h, the methanol was evaporated under vacuum. The aqueous residue was extracted with ethyl acetate (3 × 15 mL). The organic phases were washed with a saturated solution of NaHCO_3_ (3.0 mL) and then with a saturated solution of NaCl (3.0 mL). Finally, the extracts were dried over Na_2_SO_4_ and filtered. After evaporation of the solvent, the reaction crude was purified by chromatographic column on silica gel (eluent in gradient: CH_2_Cl_2_/CH_3_OH from 9.9/0.1 to 9.5/0.5). The isolated product was characterized by ^1^H and ^13^C NMR and identified as HTyr (yellow oil, yield: 40%). NMR spectra were according to those reported in the literature [45].

For the synthesis of HTyr-OL, HTyr (154 mg, 1.0 mmol) was solubilized in DMC (3.0 mL) at room temperature. Then, oleyl chloride was added (361 mg, 1.2 mmol) and the mixture was kept under stirring. After 24 h, DMC was evaporated under vacuum and the residue was solubilized with ethyl acetate (20 mL); then, a saturated solution of NaCl was added (10 mL). After the extraction with ethyl acetate (3 × 20 mL), the combined organic phases were washed with a saturated solution of NaCl (20 mL), dried over Na_2_SO_4_, and filtered. After evaporation of the solvent, the reaction crude was purified by chromatographic column on silica gel (eluent in gradient: CH_2_Cl_2_/CH_3_OH from 9.8/0.2 to 9.0/1.0). The isolated product was characterized by ^1^H and ^13^C NMR and identified as HTyr-OL (colourless oil, yield: 60%) by comparison with the literature data [31,43].

### 4.4. Cell Culturing

To evaluate the effect of HTyr-OL, HTyr and OA on cell viability, two different cell lines, human neuroblastoma cells (SH-SY5Y, ATCC^®^ CRL-2266) and normal breast epithelial cell (MCF10A. ATCC^®^ CRL-10317™) were used. The SH-SY5Y cells were cultured in DMEM F-12 supplemented with 10% Fetal Bovine Serum (FBS), 2 mM glutamine and 100 U penicillin/0.1 mg/mL streptomycin. The MCF10A cells were maintained in DMEM F-12 supplemented with 100 ng/mL cholera toxin, 20 ng/mL epidermal growth factor (EGF), 0.01 mg/mL insulin, 500 ng/mL hydrocortisone, 5% Horse Serum (HS), 2 mM glutamine and 100 U penicillin/0.1 mg/mL streptomycin; before being seeded for the viability assay, the culture medium was deprived of EGF and the HS was reduced to 2%. All the cell lines were maintained at 37 °C in a humidified 5% CO_2_ condition. All experiments were performed with the cells in their logarithmic growth phase.

### 4.5. Cytotoxicity and Anti-Proliferative Assay

The cytotoxicity and anti-proliferative activities were performed using the MTT assay, as described by Ovidi et al., 2020 [68]. The cells were seeded in 96 flat-bottomed tissue culture plate at seeding concentrations of 2 × 10^5^ cells/mL for SH-SY5Y cells, and 1 × 10^5^ cells/mL for the MCF10A cells. The plates were incubated in a humidified incubator with 5% CO_2_ at 37° C for 24 h to allow the cells to attach to the bottom of the wells. Subsequently, 10 two-fold different concentrations were added to the media, from 200 to 0.39 µM of HTyr-OL, HTyr, and OA. DMSO was used as solvent control and vinblastine as positive control. The cells were treated for 72 h for the dose-dependent cytotoxic activity and for 24, 48, and 72 h for the dose and time-dependent anti-proliferative activity. Following each treatment, the media containing the compounds were aspirated and 100 mL of 0.5 mg/mL of MTT in culture medium was added. After 3 h, the MTT solution in each well was removed, and 100 mL of DMSO was added to solubilize the purple-coloured formazan product. The absorbance was read at 595 nm using a microplate reader. The absorbance reading was corrected to background absorbance, and average reading was taken from three independent experiments. The cell viability in percentage was calculated as follows:

$$\mathrm{Cell}\;\mathrm{viability}\;\%\;=\frac{\mathrm{Average}\;\mathrm{absorbance}\;\mathrm{of}\;\mathrm{treated}\;\mathrm{cells}}{\mathrm{Average}\;\mathrm{absorbance}\;\mathrm{of}\;\mathrm{untreated}\;\mathrm{cells}\;\left(\mathrm{Ctrl}\right)}\times 100$$

The results were analysed using GraphPad Prism version 8.0.2 for Windows (GraphPad Software, La Jolla, CA, USA). The half-maximal concentration (EC_50_) for each biological replicate was determined using a constructed log–response curve. Overall EC_50_ was calculated from three independent biological replicates.

### 4.6. Apoptosis Detection by Flow Cytometry

Annexin V-FITC and PI apoptosis kit (eBioscience™) was used to perform the apoptosis detection. A total of 5 × 10^5^ SH-SY5Y cells /mL were seeded into a six well plate and, after 24 h of incubation, they were treated with HTyr-OL, HTyr and OA at their respective EC_50_ values. Cells grown in media containing DMSO 0.001%, corresponding to the higher percentage applied, were used as solvent control (Ctrl), and cells treated with vinblastine were used as apoptotic control (VBL). Cells emitting fluorescence were analysed by flow cytometry after 24 h of treatment; the cells were stained with Annexin V-FITC conjugate and propidium iodide. The population plots (dot plot), obtained by acquiring 1 × 10^4^ events of each sample, were used to define the percentage of live cells (L), early apoptotic cells (EA), late apoptotic cells (LA), and necrotic cells (N), on the basis of the florescent signal emitted by each individual event in reference to the axes; Annexin V-FITC green fluorescence in abscissa, vs. PE red fluorescence in ordinate. The experiments were repeated three times.

### 4.7. Scanning Electron Microscopy

For scanning electron microscope analysis, SH-SY5Y cells were seeded at a density of 3 × 10^5^ cells/mL on a six-well plate and incubated for 24 h under appropriate culture conditions. After treatment with EC_50_ concentrations of each compound tested (VBL and Ctrl were also added as positive and negative controls, respectively) for 24 h, the cells were collected in tubes, washed with PBS and fixed with 4% paraformaldehyde and 5% glutaraldehyde, pH 7.2, in 0.1 M cacodylate buffer for 1 h at 4 °C [69]. After rinsing the samples in the same buffer, they were post-fixed in 1% osmium tetroxide in a cacodylate buffer for 1 h at 4 °C. After the samples were washed twice in the same buffer, they were dehydrated in a graded ethanol series. The samples were then dried with a critical point dryer using CO_2_ in a Balzers Union CPD 020, coated with gold in a Balzers MED 010 unit and observed under a JEOL JSM 5200 electron microscope (Jeol Ltd., Tokyo, Japan).

### 4.8. Detection of Caspase-3 Activated by Western Blot

SH-SY5Y cells were seeded at 1 × 10^6^ cells/mL density into a Petri dish and incubated for 24 h. After treatments with HTyr, HTyr-OL, OA, and vinblastine at their respective EC_50_ concentrations, cells were lysed with NP40 buffer on ice for 30 min. Protein concentrations were determined by the Bradford test. Samples were run on a 10% SDS-PAGE and then electrotransferred onto a pure nitrocellulose membrane (0.45 µm). The membrane was blocked with 5% bovine serum albumin and immunoblotted with cleaved caspase-3 rabbit mAb and β-actin mouse mAb (Cell Signaling Technology Europe B.V., Leiden, The Netherlands). Anti-rabbit IgG (H + L) and anti-mouse IgG (H + L) HRP conjugate antibodies (Promega, Madison, WI, USA) were used to detect the corresponding bands by acquiring images by ChemiDoc XRS+ System (Bio Rad, Segrate, Italy) [70]. Acquired pictures were quantitatively evaluated using the ImageJ program version, ImageJ 1.48v-Wayne Rasband, National Institute of Health, Bethesda, MD, USA.

### 4.9. Statystical Analysis

The data from three independent experiments on cytotoxicity, anti-proliferative activity, and apoptosis rate obtained by flow cytometry were used and expressed as means ± SD. Statistical analysis was performed using one-way ANOVA test with Stat-Plus software (AnalystSoft VC 2009, Walnut, CA, USA), with the threshold of significance set at *p*-value < 0.05.

### 4.10. Proteomic Analysis

#### 4.10.1. Preparation of Cell Culture Samples

Cells lysis of SH-SY5Y human neuroblastoma cell was performed using a solution of 1% NP40. The proteins were quantified using BCA assay following manufacturers’ instructions. A total of 100 µg of total proteins for each sample was reduced with dithiothreitol (DTT), and consequently alkylated with iodoacetamide (IAA). Then, protein precipitation was performed using a solution of chloroform/methanol (1:4). The protein pellet was resuspended in a solution of 6 M urea. Digestion was performed by adding trypsin (enzyme-to-protein ratio of 1:50) and incubated at 37 °C overnight. After trypsin digestion, all reaction mixtures were acidified with 1% FA in order to inhibit any remaining enzyme activity

#### 4.10.2. Proteomic Analysis

Using OASIS cartridges (Waters), digested samples were desalted, brought to dryness, and reconstituted in 0.1% formic acid in water in order to obtain a concentration of 1 ug/ul. LC-MS/MS analyses were performed using Q Exactive™ HF-X mass spectrometer (Thermo Scientific). Peptide separation was carried out at 35 °C using a PepMap TM RSLC C18 column, 75 µm × 50 cm, 2 μm, and 100 Å at a flow rate of 250 nL/min (Thermo Scientific). The mobile phases A and B used for the analysis were 0.1% formic acid in water and 0.1% formic acid in 80% acetonitrile, respectively. The gradient started with 5% of B, which was kept constant for 5 min. Then, the organic phase was increased up to 36% in 135 min and later was raised up to 90%, kept constant for 11 min and then returned to the initial conditions. These experiments were performed using a data dependent acquisition (DDA) setting to select the “top twelve” most-abundant ions for MS/MS analysis. Proteomic analysis was performed using a Label Free Quantification (LFQ) method. Protein identification was performed using Proteome Discoverer 2.5 (Thermo Scientific) and Sequest algorithm. The peptide spectra were matched against Homo sapiens database downloaded from Uniprot (total proteins: 20,626). The analysis was based on at least two unique peptides with a minimum length of seven amino acids and a false discovery rate of 0.01, which was applied to both peptide and protein levels.

#### 4.10.3. Bioinformatics Analysis

For a functional annotation analysis, the protein identifiers were associated to their related Gene Ontology (GO) terms [71], overcoming the redundancy in terminology for “molecular function”, “biological process”, and “cellular component” [72]. A *p*-value < 0.05 was used as a threshold to show over-representation of a specific GO term. FunRich 3.1.3 [73] and ClueGO v2.5.6 (Cytoscape plugin) [74] were applied in this study for Venn diagram representations, for confirming GO results and for a graphical description of enrichment and network analysis. String version 11.0 was used to verify the shell of interactors of specific proteins [75]. The default peak-picking settings were used to process the raw MS files in MaxQuant [76] (version 1.6.1.0) and its integrated search engine Andromeda [77]. MaxQuant automatically aligned the runs. Perseus (version 1.6.1.1) was successively employed for processing of MaxQuant result files. The Label Free Quantification (LFQ) intensities of proteins were imported and transformed to logarithmic scale with base two. The missing values were replaced (imputed) by random numbers drawn from a normal distribution of 1.8 standard deviation down shift and with a width of 0.3 of each sample. The protein quantification and calculation of statistical significance was carried out using two-tailed Student’s t-test and error correction (*p*-value < 0.05) with the method of Benjamini–Hochberg. Moreover, a volcano plot, showing a summary distribution of differentially expressed proteins (DEPs) between Control (Ctrl) and Hydroxytyrosol oleate (HTyr-OL) samples, was calculated. The volcano plot is an easy-to-interpret scatter plot that arranges values along dimensions of biological (difference Ctrl and HTyr-OL) and statistical (log10 *p*-value) significance. The proteins located on the upper left region and the upper right region (S0 = 2, FDR < 0.05) are differentially expressed.

## 5. Conclusions

HTyr-OL is a lipophilic HTyr derivative found in EVOO and olive oil by-products that can be synthesized in the laboratory with a good yield and high degree purity. It appears as a potential bioactive compound showing the catecholic moiety of HTyr combined with the monounsaturated chain of OA. Despite this potentiality, to date, HTyr-OL’s biological activities have been rarely studied. Due to this gap in the literature, this study, for the first time, evaluated and demonstrated the in vitro cytotoxic, anti-proliferative, and apoptotic inducing activity of HTyr-OL against human SH-SY5Y neuroblastoma cells. Flow cytometry, Western blot and electron microscopy analyses showed that the biological activity of HTyr-OL is caused by a mechanism of cell death by apoptosis, an effect revealed at doses even four times lower than those of HTyr. Proteomic analysis supported the experimental evidence through the identification of the downregulation of cell proliferation and the up-regulation of the extrinsic apoptotic signaling pathway, demonstrating the potential role of HTyr-OL as a chemopreventive agent.

## Figures and Tables

**Table 1:** HTyr-OL, HTyr, and OA EC_50_ values against SH-SY5Y and MCF10A cells.

	EC_50_ (µM Mean ± SD)	
Compound	SH-SY5Y	MCF10A
HTyr-OL	26.35 ± 3.75 ^a^	>200
HTyr	114.02 ± 1.69	>200
OA	35.93 ± 3.57 ^b^	>200

^a^*p* < 0.05 significant differences compared with HTyr and OA treated groups. ^b^ *p* < 0.05 significant differences compared with HTyr and HTyr-OL treated groups.

**Table 2:** HTyr-OL, HTyr, and OA EC_50_ values against SH-SY5Y after 24, 48 and 72 h of treatment.

EC_50_ (µM Mean ± SD)			
Compound	24 h	48 h	72 h
HTyr-OL	48.97 ± 9.04 ^a^	27.76 ± 2.31 ^b^	26.35 ± 3.75 ^b^
HTyr	150.13 ± 7.89	113.59 ± 7.87	114.02 ± 1.69
OA	36.91 ± 4.93 ^a^	38.64 ± 5.28 ^c^	35.93 ± 3.57 ^c^

^a^*p*-value < 0.05 significant differences compared with HTyr treated group. ^b^ *p*-value < 0.05 significant differences compared with HTyr and OA treated groups. ^c^ *p*-value < 0.05 significant differences compared with HTyr and HTyr-OL treated groups.

**Table 3:** Apoptotic rate percentages obtained from the sum of the percentages of EA and LA cells. The values are expressed as % ± standard error mean. *p*-value < 0.05 significant differences compared with Ctrl group.

Compound	Apoptotic Rate %
HTyr-OL	52.36 ± 4.31
HTyr	79.64 ± 8.83
OA	49.91 ± 6.94
Ctrl	22.25 ± 2.69
VBL	74.24 ± 6.29

## Data Availability

Not applicable.

## Supplementary Materials

The supporting information can be downloaded at: https://www.mdpi.com/article/10.3390/ijms232012348/s1.

## Sample Availability

Samples of the compounds are available from the authors.
