# Supplementary material for: In Vitro Anti-Proliferative and Apoptotic Effects of Hydroxytyrosyl Oleate on SH-SY5Y Human Neuroblastoma Cells
Source: Int J Mol Sci. 2022 Oct 15;23(20):12348. doi: 10.3390/ijms232012348 (PMC9604296; doi:10.3390/ijms232012348)
Supplement: Supplementary file 1 [file ijms-23-12348-s001.zip › ijms-1924031-supplementary.pdf]

Table S1: Volcano Plot.							
**Significant**	**=-LOG(P-value)**	**Difference**	**Accession**	**ID**	**Description**	**Gene**	**Volcano plot results**
+	2,99	3,80	O00458	IFRD1_HUMAN	Interferon-relateddevelopmentalregulator1	IFRD1	up in HTyrol
+	5,52	3,58	O15240	VGF_HUMAN	NeurosecretoryproteinVGF	VGF	up in HTyrol
+	2,91	-2,92	O60343	TBCD4_HUMAN	TBC1domainfamilymember4	TBC1D4	down in HTyrOL
+	2,16	3,10	O75367	H2AY_HUMAN	Corehistonemacro-H2A.1	MACROH2A1	up in HTyrol
+	4,19	-3,44	O75376	NCOR1_HUMAN	Nuclearreceptorcorepressor1	NCOR1	down in HTyrOL
+	2,62	3,52	O76080	ZFAN5_HUMAN	AN1-typezincfingerprotein5	ZFAND5	up in HTyrol
+	4,45	2,84	O95817	BAG3_HUMAN	BAGfamilymolecularchaperoneregulator3	BAG3	up in HTyrol
+	2,74	5,02	P09601	HMOX1_HUMAN	Hemeoxygenase1	HMOX1	up in HTyrol
+	3,14	3,81	P10909	CLUS_HUMAN	Clusterin	CLU	up in HTyrol
+	3,25	-2,30	P51553	IDH3G_HUMAN	Isocitratedehydrogenase[NAD]subunitgamma,mitochondrial	IDH3G	down in HTyrOL
+	3,28	-2,62	P56270	MAZ_HUMAN	Myc-associatedzincfingerprotein	MAZ	down in HTyrOL
+	2,38	3,10	P62805	H4_HUMAN	HistoneH4	H4-16	up in HTyrol
+	3,86	2,04	Q04446	GLGB_HUMAN	1,4-alpha-glucan-branchingenzyme	GBE1	up in HTyrol
+	3,28	2,16	Q13501	SQSTM_HUMAN	Sequestosome-1	SQSTM1	up in HTyrol
+	2,80	-2,21	Q13547	HDAC1_HUMAN	Histonedeacetylase1	HDAC1	down in HTyrOL
+	3,42	3,35	Q14517	FAT1_HUMAN	ProtocadherinFat1	FAT1	up in HTyrol
+	3,56	-2,22	Q14684	RRP1B_HUMAN	RibosomalRNAprocessingprotein1homologB	RRP1B	down in HTyrOL
+	3,44	5,31	Q15011	HERP1_HUMAN	Homocysteine-responsiveendoplasmicreticulum-residentubiquitin-likedomainmember1protein	HERPUD1	up in HTyrol
+	2,07	2,86	Q16777	H2A2C_HUMAN	HistoneH2Atype2-C	H2AC20	up in HTyrol
+	1,93	3,54	Q16778	H2B2E_HUMAN	HistoneH2Btype2-E	H2BC21	up in HTyrol
+	2,35	-2,44	Q3ZCT1	ZN260_HUMAN	Zincfingerprotein260	ZNF260	down in HTyrOL
+	2,41	3,93	Q5TEC6	H3PS2_HUMAN	HistoneHIST2H3PS2	H3-2	up in HTyrol
+	2,68	-2,41	Q6IN84	MRM1_HUMAN	rRNAmethyltransferase1,mitochondrial	MRM1	down in HTyrOL
+	4,19	-2,33	Q8N2U9	S66A2_HUMAN	Solutecarrierfamily66member2	SLC66A2	down in HTyrOL
+	2,42	-2,69	Q8TCC3	RM30_HUMAN	39SribosomalproteinL30,mitochondrial	MRPL30	down in HTyrOL
+	2,07	-2,88	Q8WVB6	CTF18_HUMAN	Chromosometransmissionfidelityprotein18homolog	CHTF18	down in HTyrOL
+	2,79	-2,63	Q969E4	TCAL3_HUMAN	TranscriptionelongationfactorAprotein-like3	TCEAL3	down in HTyrOL
+	3,32	-2,42	Q96G46	DUS3L_HUMAN	tRNA-dihydrouridine(47)synthase[NAD(P)(+)]-like	DUS3L	down in HTyrOL
+	2,30	-3,02	Q96MX3	ZNF48_HUMAN	Zincfingerprotein48	ZNF48	down in HTyrOL
+	2,72	-3,15	Q96NE9	FRMD6_HUMAN	FERMdomain-containingprotein6	FRMD6	down in HTyrOL
+	3,42	-2,40	Q96QC0	PP1RA_HUMAN	Serine/threonine-proteinphosphatase1regulatorysubunit10	PPP1R10	down in HTyrOL

+	2,64	3,07	Q9BYN0	SRXN1_HUMAN	Sulfiredoxin-1	SRXN1	up in HTyrol
+	2,62	3,44	Q9BZQ8	NIBA1_HUMAN	ProteinNiban1	NIBAN1	up in HTyrol
+	3,18	-2,19	Q9NX58	LYAR_HUMAN	Cellgrowth-regulatingnucleolarprotein	LYAR	down in HTyrOL
+	2,51	-2,68	Q9NYP7	ELOV5_HUMAN	Elongationofverylongchainfattyacidsprotein5	ELOVL5	down in HTyrOL
+	3,46	-2,63	Q9UKB3	DJC12_HUMAN	DnaJhomologsubfamilyCmember12	DNAJC12	down in HTyrOL
+	3,17	-4,16	Q9UNS1	TIM_HUMAN	Proteintimelesshomolog	TIMELESS	down in HTyrOL
+	3,27	-3,10	Q9Y6X9	MORC2_HUMAN	ATPaseMORC2	MORC2	down in HTyrOL
